# Light-Induced Movements of Chloroplasts and Nuclei Are Regulated in Both Cp-Actin-Filament-Dependent and -Independent Manners in *Arabidopsis thaliana*

**DOI:** 10.1371/journal.pone.0157429

**Published:** 2016-06-16

**Authors:** Noriyuki Suetsugu, Takeshi Higa, Eiji Gotoh, Masamitsu Wada

**Affiliations:** 1 Department of Biology, Faculty of Sciences, Kyushu University, Fukuoka, Japan; 2 Department of Agriculture, Kyushu University, Fukuoka, Japan; University of Texas at Austin, UNITED STATES

## Abstract

Light-induced chloroplast movement and attachment to the plasma membrane are dependent on actin filaments. In *Arabidopsis thaliana*, the short actin filaments on the chloroplast envelope, cp-actin filaments, are essential for chloroplast movement and positioning. Furthermore, cp-actin-filament-mediated chloroplast movement is necessary for the strong-light-induced nuclear avoidance response. The proteins CHLOROPLAST UNUSUAL POSITIONING 1 (CHUP1), KINESIN-LIKE PROTEIN FOR ACTIN-BASED CHLOROPLAST MOVEMENT 1 (KAC1) and KAC2 are required for the generation and/or maintenance of cp-actin filaments in *Arabidopsis*. In land plants, CHUP1 and KAC family proteins play pivotal roles in the proper movement of chloroplasts and their attachment to the plasma membrane. Here, we report similar but distinct phenotypes in chloroplast and nuclear photorelocation movements between *chup1* and *kac1kac2* mutants. Measurement of chloroplast photorelocation movement indicated that *kac1kac2*, but not *chup1*, exhibited a clear strong-light-induced increase in leaf transmittance changes. The chloroplast movement in *kac1kac2* depended on phototropin 2, CHUP1 and two other regulators for cp-actin filaments, PLASTID MOVEMENT IMPAIRED 1 and THRUMIN 1. Furthermore, *kac1kac2* retained a weak but significant nuclear avoidance response although *chup1* displayed a severe defect in the nuclear avoidance response. The *kac1kac2chup1* triple mutant was completely defective in both chloroplast and nuclear avoidance responses. These results indicate that CHUP1 and the KACs function somewhat independently, but interdependently mediate both chloroplast and nuclear photorelocation movements.

## Introduction

Organelle movement is essential for many cellular activities and thus needs to be tightly regulated [1, 2]. Because land plants are sessile organisms, the organelle movements should be appropriately regulated by environmental signals, such as light. Among plant organelles, chloroplasts change their position in response to light (chloroplast photorelocation movement). Chloroplasts move towards weak light to capture light efficiently (the accumulation response). Conversely, chloroplasts escape from strong light and move to a position where light absorption is minimized (the avoidance response) [3, 4]. Phototropin (phot) is the blue light receptor for chloroplast photorelocation movement. In *Arabidopsis thaliana*, two phototropins, phot1 and phot2, redundantly mediate the accumulation response, and phot2 primarily regulates the avoidance response [5–7].

In general, organelle movement is dependent on actin filaments in land plants. The movements of most organelles, such as mitochondria, Golgi bodies, and endoplasmic reticulum, rely on the cytoplasmic actin cables and motor protein myosins. However, chloroplast movement is mediated by specialized chloroplast-actin (cp-actin) filaments and is likely independent of myosins [8]. Cp-actin filaments are localized mostly on the chloroplast periphery, between chloroplasts and the plasma membrane [9, 10]. Blue light regulates the amount, and the positions, of cp-actin filaments. Strong blue light induces the disappearance of cp-actin filaments by severing the filaments in a phot2-dependent manner [10], and then cp-actin filaments are polymerized at the future front regions of the chloroplasts [9–11]. Weak blue light induces the polymerization of cp-actin filaments without the detectable severing [9, 10]. The blue-light-induced reorganization of cp-actin filaments is mediated by phototropins [9–11], and phot2 is a master regulator of the strong-light-induced response [10]. Other components are involved in the light regulation of cp-actin filaments. A J-domain protein, J-DOMAIN PROTEIN REQUIRED FOR CHLOROPLAST ACCUMULATION RESPONSE 1 (JAC1), plays a particularly important role in the accumulation response [12] and is essential for the reorganization of cp-actin filaments during the weak-light-induced accumulation response [11]. Mutant plants of two interacting coiled-coil proteins, WEAK CHLOROPLAST MOVEMENT UNDER BLUE LIGHT 1 (WEB1) and PLASTID MOVEMENT IMPAIRED 2 (PMI2), are defective in the blue-light-induced reorganization of cp-actin filaments during the avoidance response, and thus, *web1* and *pmi2* mutants exhibit the attenuated chloroplast avoidance response [13, 14]. A C2 domain protein, PLASTID MOVEMENT IMPAIRED 1 (PMI1), is essential for chloroplast movement, and the *pmi1* mutant is severely defective in chloroplast photorelocation movement [15]. The cp-actin filaments are labile in *pmi1*, indicating that PMI1 is necessary for the stability of cp-actin filaments [16]. THRUMIN1 (THRUM1) is an actin-binding and -bundling protein [17]. The *thrum1* mutants are partially defective in chloroplast movement and are severely impaired in the accumulation of cp-actin filaments [10, 17].

The light-induced reorganization of cp-actin filaments was found in the fern *Adiantum capillus-veneris* [18] and the moss *Physcomitrella patens* [19], indicating that the cp-actin-filament-based chloroplast movement is conserved among land plants. Two protein families, CHLOROPLAST UNUSUAL POSITIONING1 (CHUP1) and KINESIN-LIKE PROTEIN FOR ACTIN-BASED CHLOROPLAST MOVEMENT (KAC), are indispensable for the polymerization and/or maintenance of cp-actin filaments and have conserved functions in land plants [20–25]. In *Arabidopsis*, mutants deficient in CHUP1 or two KAC proteins (KAC1 and KAC2) show severe defects in chloroplast movement and positioning [20, 21, 22, 26]. The chloroplasts of these mutant plants are detached from the plasma membrane, resulting in aberrant positioning and the absence of directional photorelocation movement because they lack detectable cp-actin filaments [9, 10, 11, 22]. CHUP1 is a multi-domain protein that consists of an N-terminal hydrophobic region, a coiled-coil domain, an actin-binding motif, a proline-rich region and a conserved C-terminal region [20]. CHUP1 is localized on the chloroplast outer envelope through the N-terminal hydrophobic region [20, 21, 27]. CHUP1 interacts with F-actin, G-actin, and profilin *in vitro* probably through the C-terminal region, which includes the actin-binding motif and the proline-rich region [20, 27]. The N-terminal coiled-coil domain serves as a dimerization domain [28] and is essential for the binding of CHUP1 to the plasma membrane [21]. KAC is a microtubule motor kinesin-like protein. Although KAC belongs to the kinesin-14 family, including minus end-directed motors with a C-terminal motor domain, no detectable microtubule motor activity was observed [22, 29]. Similar phenotypes between *chup1* and *kac1kac2* in *Arabidopsis* suggest that CHUP1 and KAC proteins coordinately mediate cp-actin-mediated chloroplast movement and positioning, although the mechanism is unknown.

The movement of nuclei is also regulated by blue light [30] and dependent on phototropins in *Arabidopsis* [31] and the fern *A*. *capillus-veneris* [32]. In *Arabidopsis*’ mesophyll and pavement cells the nuclei are situated on the cell bottom in the darkness (dark position) and move to the side walls (light position) in response to strong blue light as a result of the nuclear avoidance response [31, 33, 34]. We recently demonstrated that the nuclear avoidance response is mediated by the movement of plastids and chloroplasts attached to the nuclei in *Arabidopsis*’ pavement and mesophyll cells, respectively [34]. Using green fluorescent protein (GFP)-talin lines, the position of the nucleus and the associated actin filaments were visualized simultaneously [34]. When the side of the nucleus along the long axis is partially irradiated with strong blue light and the pattern of nuclear movements was analyzed, two types of light-induced nuclear movements are observed. One is “the avoidance movement” in which the nuclei escape from the irradiated side towards the non-irradiated side. The avoidance movement depends on cp-actin-filament-dependent movement of plastids attached to the nuclei and is induced specifically by blue light in a phot2-dependent manner [34]. The other is “the parallel movement” in which the nuclei move parallel to the actin bundles. The parallel movement is independent of plastids and is induced mostly in a photosynthesis-dependent manner [34]. Mutant analyses have indicated that the nuclear avoidance response is dependent on CHUP1 and PMI1, in addition to phot2 [16, 34]. The nuclear avoidance response in mesophyll cells is mediated by PMI1 alone, but the response in the pavement cells depends on both PMI1 and the homologous PMI1-RELATED1 (PMIR1), but not on PMIR2 [16]. However, the role of the KAC proteins in the nuclear avoidance response remained to be determined because the blue-light-induced movements of plastids and nuclei had not been analyzed in *kac1kac2* pavement cells.

To understand the role of KAC proteins, especially the relationship between KAC and other proteins, we generated multiple mutant plants between *kac1kac2* and other mutants and analyzed light-induced movement of chloroplasts and nuclei in these mutants. Here, we found clear differences in chloroplast and nuclear movements between *chup1* and *kac1kac2* in *Arabidopsis*, although previous studies suggested that CHUP1 and KAC proteins function as the same pathway [22, 24].

## Materials and Methods

### Plant materials and plant growth

*Arabidopsis* seeds (Columbia) were sown on one-third-strength Murashige and Skoog culture medium containing 1% (w/v) sucrose and 0.8% (w/v) agar. After incubation for 2 d at 4°C, the plants were cultured under white light at approximately 100 μmol m^–2^ s^–1^ under a 16/8-h light/dark cycle at 23°C in a growth chamber. Approximately 2-week-old plants were used for the analyses of chloroplast and nuclear photorelocation movements. To observe the chloroplast distribution, plants were cultured on soil (Metro Mix 350; Sun Gro, Vancouver, BC, Canada) under white light at approximately 80 μmol m^–2^ s^–1^ under a 16/8-h light/dark cycle in a growth chamber. The N7 nuclear marker line [35] was provided by the Arabidopsis Biological Stock Center. Double- and triple-mutant plants were generated by genetic crossings. Mutant lines containing the N7 nuclear marker and GFP-mouse-talin [9, 10] were generated by genetic crossings.

### Analyses of chloroplast photorelocation movements

Chloroplast photorelocation movement was examined by measuring changes in leaf transmittance as described previously [36]. The detached third leaves from 16-day-old plants were placed on 1% (w/v) gellan gum in a 96-well plate. Samples were dark-adapted for at least 1 h prior to transmittance measurements. Blue light was supplied from a blue light-emitting diode illuminator (LED-mB; EYELA). The red light transmittance was automatically measured every 2 min using a microplate reader (VersaMax; Molecular Devices). To disrupt actin filaments, the detached third leaves were treated with 10 μM latrunculin B. The inhibitor treatment was performed by floating the leaves on the inhibitor solution for 12 h.

### Observation of the chloroplast distribution

Three-week-old soil-grown plants were dark-adapted for 8 h. Then, plants were kept in the darkness for 2 additional h or irradiated with blue light at 30 μmol m^–2^ s^–1^ for 2 h. The leaves were collected and fixed as described previously [34].

### Analyses of nuclear photorelocation movements

Time-course experiments for the nuclear photorelocation movement were performed as described previously [34]. After 2-week-old plants were dark-adapted for 24 h, they were irradiated with 50-μmol m^–2^ s^–1^ blue light for 12 h. The leaves were collected and fixed at 0, 3, 6, 9, and 12 h after light irradiation, as described previously [34]. Data for wild type and *chup1* from [34] were used for comparison because those for *kac1kac2* and *chup1kac1kac2* were acquired during the same experimental period.

### Confocal laser scanning microscopy

The cp-actin filaments, nuclear movement, and the plastids attached to the nucleus were observed under a confocal microscope (SP5; Leica Microsystems) as described previously [10, 34]. Fluorescence was observed at 500–550 nm for GFP and 650–710 nm for chlorophyll autofluorescence.

## Results

### Strong-blue-light-induced changes in leaf transmittance in the *kac1kac2* double mutant

**Fig 1:**
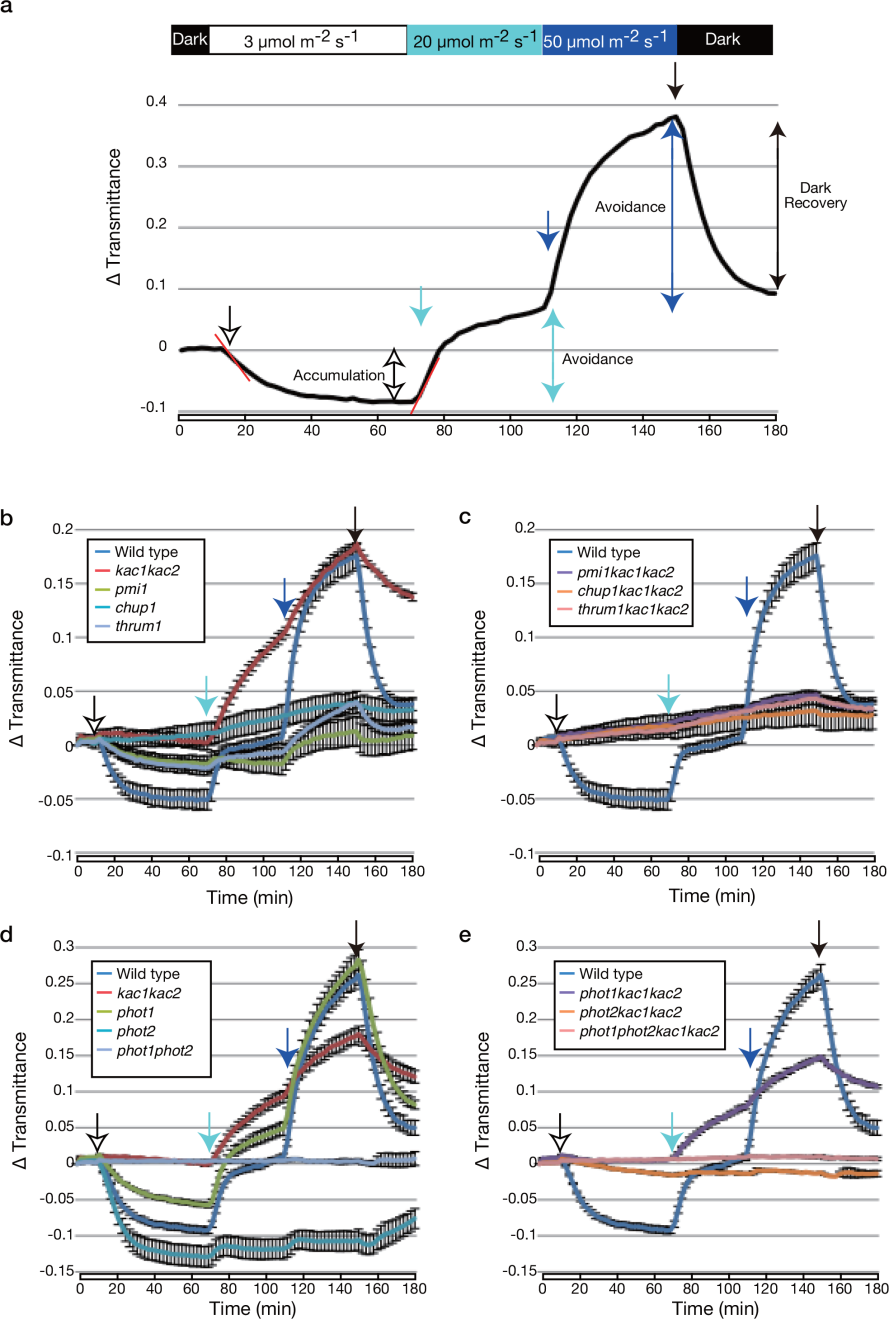
Chloroplast photorelocation movement induced by strong blue light in *kac1kac2*. **(a)** Analysis of chloroplast photorelocation movements by measuring the light-induced changes in leaf transmittance. A black trace represents a typical light-induced change in wild type under our light irradiation condition (indicated by a colored bar above a graph). Transmittance changes are indicated as ∆ transmittance (a transmittance value at the indicated time minus the transmittance at 0 min). After the measurement was performed for 10 min in darkness, samples were sequentially irradiated with continuous blue light at 3, 20, and 50 μmol m^–2^ s^–1^ for 60, 40, and 40 min, as indicated by white, sky blue, and blue arrows, respectively. The light was extinguished at 150 min (black arrow). Double-headed arrows indicate the chloroplast movements induced under the indicated light conditions. Red lines indicate the initial linear fragments (from 2 to 6 min after changes in light fluence rate) which were used for the calculation of the percentage transmittance change over 1 min (i.e., speed of leaf transmittance changes). **(b-e)** KAC-independent chloroplast movement was analyzed in *chup1*, *pmi1*, and *thrum1* backgrounds **(b, c)**, in the phototropin mutant background **(d, e)**. Changes in leaf transmittance caused by the chloroplast photorelocation movement. Mean values from three independent experiments are shown. Error bars indicate standard errors.

To further examine the defective chloroplast photorelocation movements in *chup1* and *kac1kac2*, we examined light-induced changes in leaf transmittance in these mutants (Fig 1A). This method allows the non-invasive measurement of chloroplast photorelocation movements in multiple leaf cells and layers [37], and defective chloroplast movement in various *Arabidopsis* mutants were reliably detected [9, 14, 16, 38]. As indicated in Fig 1A, in the wild type, the decrease in leaf transmittance was induced by weak blue light (3 μmol m^–2^ s^–1^; white arrow in Fig 1) as a result of the accumulation response, whereas strong blue light (20 or 50 μmol m^–2^ s^–1^; sky blue or blue arrows in Fig 1, respectively) induced an increase in leaf transmittance as a result of the avoidance response. After the light was extinguished (black arrow in Fig 1), the transmittance rapidly decreased and reached the basal level (the dark recovery response). As expected, in the *chup1* mutant, no detectable light-induced changes in leaf transmittance were detected (Fig 1B and S1 Fig). However, unexpectedly, we detected clear leaf transmittance changes in *kac1kac2*. Although weak blue light did not induce any changes in leaf transmittance, *kac1kac2* exhibited a clear increase in leaf transmittance in response to strong blue light (Fig 1B and S1 Fig), indicating that the avoidance response-like chloroplast movements still occurs in the *kac1kac2* double mutant. Furthermore, after the light was extinguished, the decrease in the transmittance was very slow compared with the wild type (Fig 1B and S1 Fig).

To reveal the regulators involved in mediating the chloroplast movement in the *kac1kac2* double mutants, we crossed various mutants defective in chloroplast photorelocation movement with *kac1kac2* double mutants and analyzed leaf transmittance changes in the resulting triple or quadruple mutant plants. *phot1kac1kac2* displayed a similar phenotype as *kac1kac2* (Fig 1D and 1E and S1 Fig; Student’s *t* test, *P >* 0.08 for *kac1kac2 vs*. *phot1kac1kac2* at 20 μmol m^–2^ s^–1^). Consistent with phot2 mainly functioning under strong blue light conditions [38], the *phot2kac1kac2* mutants were severely deficient in the strong-light-induced-increase in leaf transmittance but retained a subtle weak-light-induced decrease and strong-light-induced increase in leaf transmittance compared with *phot1phot2* (Fig 1D and 1E). These responses were phot1-dependent because *phot1phot2kac1kac2* exhibited no detectable changes in leaf transmittance irrespective of the light intensity, similar to *phot1phot2* (Fig 1D and S1 Fig; Student’s *t* test, *P >* 0.1 for *phot1phot2 vs*. *phot1phot2kac1kac2* at all fluence rates). These results indicate that phot2 is the primal photoreceptor for the strong-light-induced chloroplast movements in *kac1kac2*.

The *jac1* mutant plants were unresponsive to weak blue light but responsive to strong blue light as a result of the avoidance response [12, 14]. Although the pattern of changes in the leaf transmittance was similar between *jac1* and *kac1kac2*, the increase in the leaf transmittance in response to strong light, especially at 20 μmol m^–2^ s^–1^, was much larger in *jac1* than in *kac1kac2*. Furthermore, the *jac1kac1kac2* triple mutant plants resembled the *kac1kac2* (S1 Fig). These results indicate that the avoidance response induced in *jac1* mutant plants is largely KAC-independent and vice versa. Both *web1* and *pmi2pmi15* mutant plants were defective in the strong-light-induced avoidance response [14], and thus, only slight increases were induced in both mutants (S1 Fig). However, *web1kac1kac2* and *pmi2pmi15kac1kac2* exhibited larger increases in the transmittance change when compared with *web1* and *pmi2pmi15*, respectively (S1 Fig). The phenotypes of *web1kac1kac2* and *pmi2pmi15kac1kac2* somewhat resembled those of *kac1kac2*, indicating that the *kac1kac2* mutation suppressed the weak avoidance response in *web1* and *pmi2pmi15*, similar to the *jac1* mutation [14]. Because *jac1kac1kac2*, *web1kac1kac2*, and *pmi2pmi15kac1kac2* showed a strong-light-induced increase in transmittance similar to that of *kac1kac2*, this indicated that JAC1, WEB1, and PMI2 (and PMI15) are dispensable for the strong-light-induced chloroplast movements in *kac1kac2*.

Similar to *chup1*, *chup1kac1kac2* is defective in any chloroplast photorelocation movement (Fig 1A and 1B; Student’s *t* test, *P >* 0.1 for *chup1* vs *chup1kac1kac2* at all fluence rates). Interestingly, *pmi1kac1kac2* and *thrum1kac1kac2* synergistically impaired chloroplast photorelocation movements compared with *kac1kac2*, *pmi1*, and *thrum1*. They were similar to that of *chup1* (Fig 1E and 1F; Student’s *t* test, *P >* 0.2 for *chup1 vs*. *pmi1kac1kac2* or *thrum1kac1kac2* at all fluence rates), indicating that PMI1 and THRUM1 are required for the strong-light-induced chloroplast movements in *kac1kac2*. Table 1 summarizes the mutant phenotypes (Table 1).

**Table 1:** Summary of the data on chloroplast photorelocation movements in Fig 1 and Fig 2. Mutant phenotypes or inhibitor effect in wild type or *kac1kac2* mutant backgrounds are indicated. Ac: accumulation response, Av: avoidance response, Av (*kac*-like): the avoidance response found in *kac1kac2* mutants, -: no or severely defective responses. Here, strong blue-light-induced response in *jac1* is categorized into *kac*-like avoidance response.

light	Weak light		Strong light	
	Wild type	*kac1kac2*	Wild type	*kac1kac2*
Wild type	Ac	-	Av	Av (*kac*-like)
*phot1*	weak Ac	-	Av	Av (*kac*-like)
*phot2*	Ac	-	-	-
*phot1phot2*	-	-	-	-
*chup1*	-	-	-	-
*pmi1*	weak Ac	-	weak Av	-
*thrum1*	weak Ac	-	weak Av	-
*jac1*	-	-	Av (*kac1*-like)	Av (*kac*-like)
*web1*	Ac	-	weak Av	Av (*kac*-like)
*pmi2pmi15*	Ac	-	weak Av	Av (*kac*-like)
Latrunculin-B	-	-	-	-

### Strong-blue-light-induced changes in leaf transmittance in the *kac1kac2* double mutant depends on actin filaments

**Fig 2:**
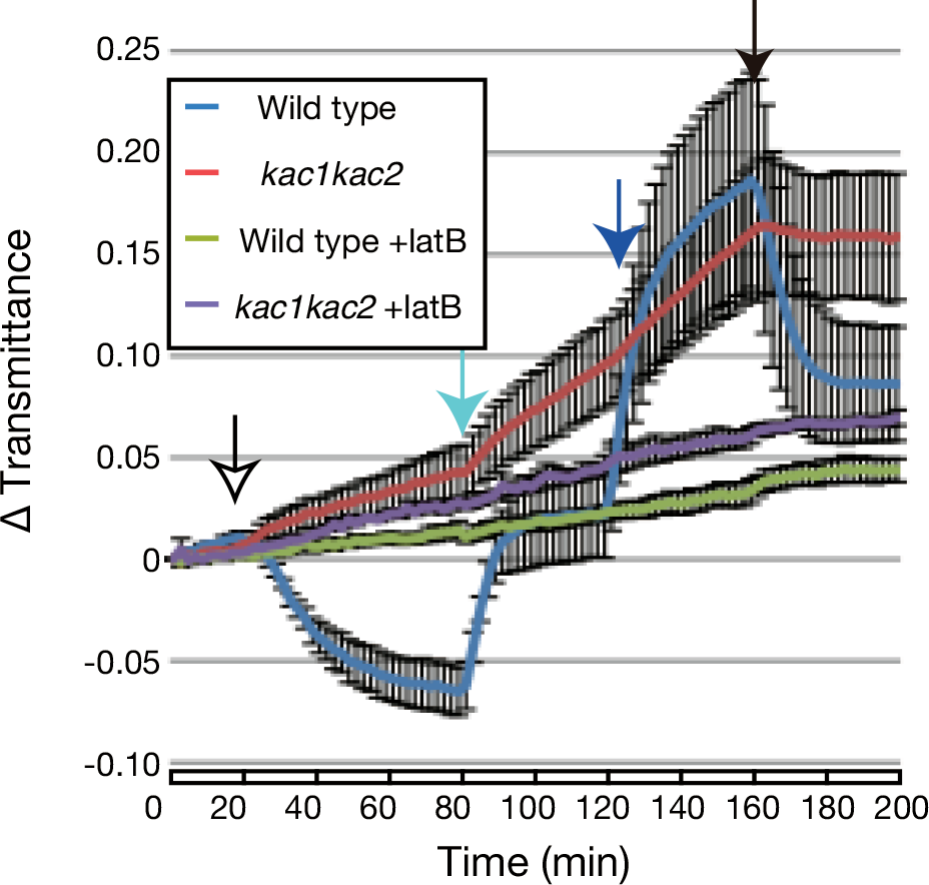
Actin filament-dependent light-induced chloroplast movement in *kac1kac2*. Chloroplast movement was analyzed in wild type and *kac1kac2*, which were treated with and without 10 μM latrunculin B. The other details are the same as in Fig 1, except there was a 20-min dark adaptation before the blue light irradiation. Mean values from four independent experiments are shown. Error bars indicate standard errors.

Our previous analyses indicated that *kac1kac2* lacks detectable cp-actin filaments [8, 22]. Thus, it is plausible that KAC-independent chloroplast movement is also independent of cp-actin filaments. To examine whether KAC-independent chloroplast movement is still actin-dependent, we investigated chloroplast photorelocation movement in wild type and *kac1kac2* mutant plants treated with the actin polymerization inhibitor latrunculin B, which inhibits blue-light-induced movements of chloroplasts and nuclei in *Arabidopsis* wild type plants [21, 33]. Treatment with latrunculin B completely abrogated any blue-light-induced changes in leaf transmittance in both wild type and *kac1kac2* mutant plants, although the leaf transmittance continued to increase in a light-independent manner (Fig 2 and Table 1). Thus, the strong-light-induced chloroplast movements in *kac1kac2* are also dependent on actin filaments.

### Subtle strong-blue-light-induced chloroplast movement from the periclinal to anticlinal walls in the *kac1kac2* double mutant

**Fig 3:**
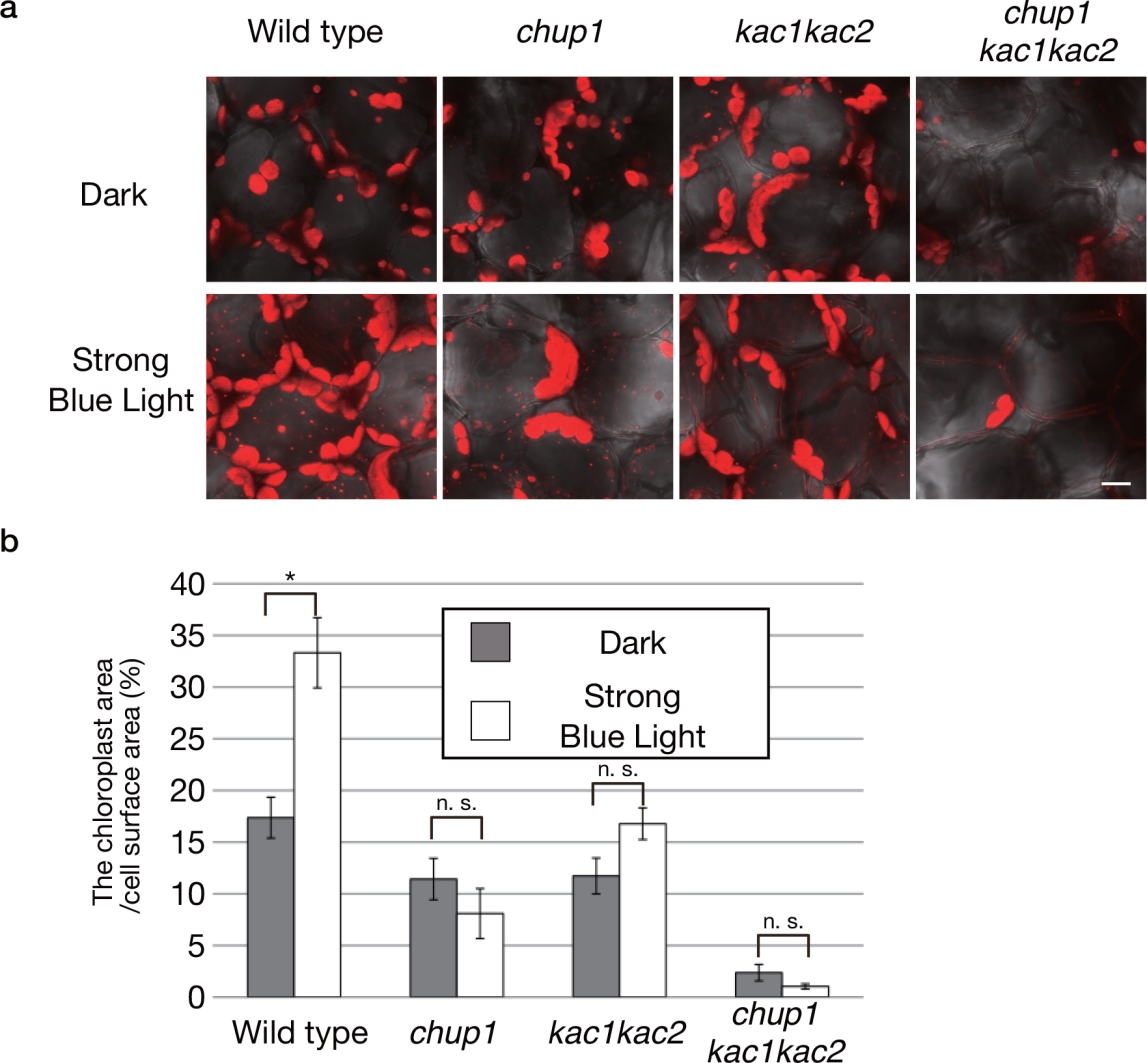
Chloroplast photorelocation movement in uppermost mesophyll cells of *kac1kac2* mutant plants. **(a)** Strong-light-induced change in the chloroplast distribution on the uppermost mesophyll cell surface. Photographs of the mesophyll cell surface in dark-adapted or strong-blue-light-irradiated plants. Bar: 10 μm. **(b)** Comparison of the ratio of the area covered with chloroplasts to the whole cell surface area. Mean values from three independent leaves are shown. Ten cells per a leaf were analyzed. Error bars indicate standard errors. An asterisk indicates the statistically significant differences, assessed by Student’s t-test. n. s. indicates the statistically insignificant difference.

Very few chloroplasts are localized on the mesophyll cell surface even under weak light conditions, when chloroplasts cover the cell surface in the wild type as a result of the accumulation response [22]. Furthermore, experiments with microbeam irradiation revealed no detectable directional movements in response to the strong blue light in *kac1kac2* [22]. However, when we closely observed the chloroplast distribution in dark-adapted or strong-light-irradiated plants, we noticed slight light-induced changes in the chloroplast distribution in *kac1kac2* but not in *chup1* or *chup1kac1kac2* (Fig 3). In the wild type, some chloroplasts were situated on the cell surface and anticlinal cell walls in the darkness, whereas chloroplasts covered the whole anticlinal wall surface under the strong light condition (Fig 3A), resulting in an increase in the ratio of the area occupied by chloroplasts (called the “chloroplast area”) to the area of the whole cell surface in the dark (Fig 3B; Student’s *t* test, *P <* 0.05 for dark *vs*. strong light). In the darkness, the chloroplast area was slightly smaller in both *chup1* and *kac1kac2* than in the wild type, and in response to the strong blue light the area did not change in *chup1* (Fig 3B; Student’s *t* test, *P >* 0.3 for dark *vs*. strong light). Statistically insignificant, but the area slightly increased in *kac1kac2* (Fig 3B; Student’s *t* test, 0.05 *< P <* 0.1 for dark *vs*. strong light). Most chloroplasts of the *chup1kac1kac2* mutant did not localize on the cell surface. The chloroplast area was much smaller than in *chup1* or *kac1kac2* and did not change in response to light (Fig 3B; Student’s *t* test, *P >* 0.1 for dark *vs*. strong light). Thus, chloroplasts in *kac1kac2* retained their movement from the periclinal to anticlinal walls. Although we observed only subtle light-induced changes in the chloroplast distribution on the surface of the top mesophyll cell layer in *kac1kac2*, the chloroplast movements in the multiple layers of palisade and sponge cells might result in the strong-light-induced changes in leaf transmittance in *kac1kac2*.

### KAC proteins and CHUP1 redundantly mediate the nuclear avoidance response in pavement cells

**Fig 4:**
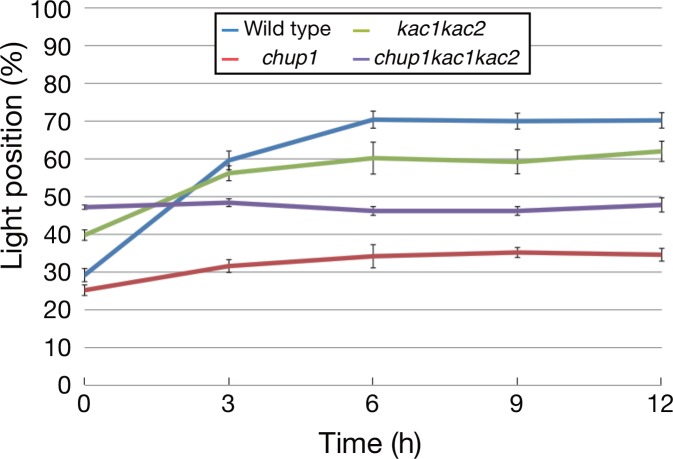
Roles of KACs and CHUP1 in the nuclear avoidance response in pavement cells. The time-course analysis of the nuclear avoidance response was performed in indicated lines. Dark-adapted plants were irradiated with strong blue light (50 μmol m^–2^ s^–1^). The percentage of nuclei in the light position is indicated. Values shown are means ± SD. Each data point was obtained from five leaves with 100 cells observed in each leaf.

In approximately 70% of wild type pavement cells, nuclei were situated on the cell bottom in darkness. A strong blue light induced nuclear movements from the cell bottom to the anticlinal walls, and nuclei were in the light position in 70% of the pavement cells after 6 h. Thus, a 40% increase in nuclei in the light position occurred in response to the strong blue light in wild type (Fig 4). Consistent with our previous results [34], *chup1* mutants were severely impaired in the nuclear avoidance response and exhibited a subtle increase in the nuclei in the light position. However, nuclei in only 35% of the cells were in the light position even after 12 h (Fig 4). The *kac1kac2* mutant showed a slight defect in dark positioning and nuclei were positioned on the cell bottom in 60% of the pavement cells, which was 10% less than in wild type (Fig 4). Furthermore, the avoidance response was partially defective in *kac1kac2*. In response to a strong blue light, only a 20% increase in nuclei in the light position occurred, and the nuclei were in the light position in 60% of the pavement cells even after 12 h (Fig 4). Thus, *kac1kac2* is partially defective in the nuclear avoidance response although it exhibited much weaker defects than *chup1*, which is similar to the chloroplast photorelocation movement, as mentioned above. The *chup1kac1kac2* triple mutant was completely impaired in the nuclear avoidance response and did not display any light-induced changes in the nuclear position. Interestingly, 45–50% of nuclei were in the light position irrespective of light conditions (Fig 4).

**Fig 5:**
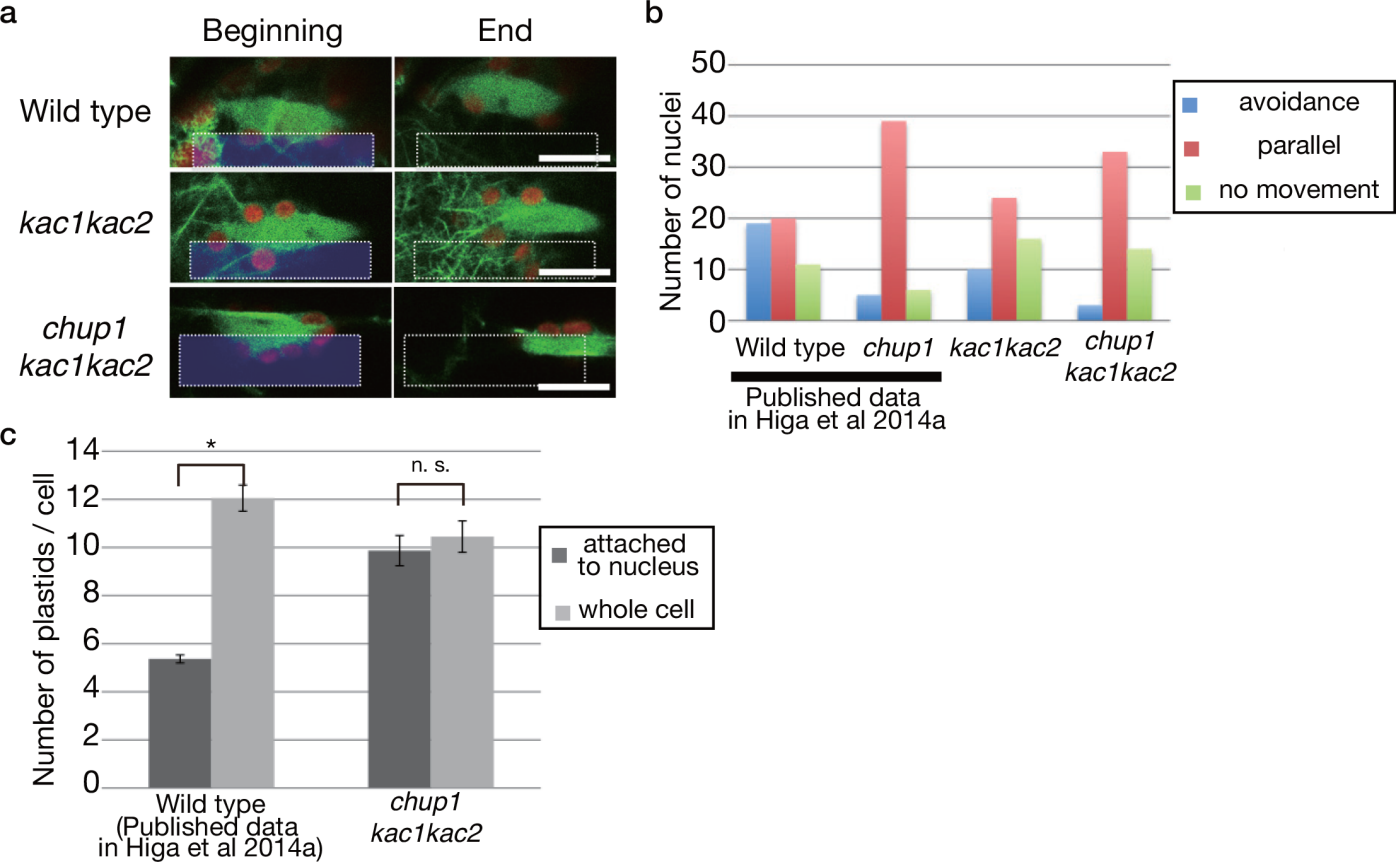
Distinct functions of KACs and CHUP1 in the nuclear photorelocation movement. **(a)** Nuclear photorelocation movement induced using a microbeam. The area irradiated with blue light is indicated as squares with dotted lines. Bar: 20 μm. **(b)** Number of nuclei showing avoidance (blue), parallel (red), or no movement (light green) in the indicated mutants. Data for wild type and *chup1* mutant plants from Higa et al. (2014a) are shown for comparison. **(c)** The number of plastids attached to a nucleus in pavement cells. Data were obtained from three leaves, with 20 cells observed in each leaf, and are presented as means ± SD. An asterisk indicates the statistically significant differences, assessed by Student’s t-test. n. s. indicates the statistically insignificant difference.

To further analyze the light-induced nuclear movement, the side of the nucleus along the long axis was irradiated with strong a blue light microbeam in lines expressing GFP-talin (see Introduction) [34]. In wild type, approximately 80% of the nuclei exhibited light-induced movement and about 40% of the nuclei showed the avoidance movement (Fig 5A; data from [34]). The *chup1* mutant had severely attenuated the avoidance movements and the parallel movement was prominent in *chup1*, indicating that cp-actins existed on the plastids attached to the nuclei, which is essential for the avoidance response and to suppress parallel movements (Fig 5A; data from [34]; chi-square test, *P >* 0.1 for wild type *vs*. *chup1*). The *kac1kac2* was similar to wild type (chi-square test, *P <* 0.0005 for wild type *vs*. *kac1kac2*), but only approximately 20% of the nuclei showed the avoidance movement (Fig 5A and 5B). This subtle defect in the avoidance movement explains the partial defects in the nuclear avoidance response in *kac1kac2*. The *chup1kac1kac2* triple mutant plants were similar to the *chup1* plants in that the parallel movement was dominant and almost no avoidance movement was observed (Fig 5A and 5B; chi-square test, *P >* 0.1 for *chup1 vs*. *chup1kac1kac2*). However, compared with *chup1*, the proportion of parallel movement was reduced and the absence of movement was increased.

Approximately half of the plastids were attached to the nucleus in wild type pavement cells and more plastids were associated with the nucleus in *chup1* mutant pavement cells [34]. The relative adhesive power between plastids and the plasma membrane may be dependent on cp-actin filaments, which determined the number of plastids attached to the nucleus [34]. Interestingly, all of the plastids were attached to the nucleus in pavement cells of *chup1kac1kac2* triple mutant plants (Fig 5C; Student’s *t* test, *P >* 0.5 for attached to nucleus *vs*. whole cell), indicating that the *chup1kac1kac2* triple mutant plants are completely defective in the attachment of plastids to the plasma membrane.

## Discussion

### Distinct functions of CHUP1 and KAC proteins in chloroplast photorelocation movement

In this report, we found distinct phenotypes for *chup1* and *kac1kac2* with regard to chloroplast photorelocation movement and the nuclear avoidance response. Detailed microbeam experiments indicated that *kac1kac2* mutants were totally defective in the “directional” chloroplast photorelocation movement [22], similar to *chup1* mutant plants [9]. The biased localization of cp-actin filaments at the front region of the chloroplast is essential for the directional chloroplast movement, and the greater difference in the amount of cp-actin filaments between the front and rear regions is necessary for efficient chloroplast movement [9, 10, 11, 22]. Together with the reduced attachment of chloroplasts from the plasma membrane, these phenotypes are largely attributable to the lack of cp-actin filaments in both mutants [9,10,11,22]. The measurement of leaf transmittance changes revealed that a clear chloroplast movement in response to strong blue light was observed in *kac1kac2*, but not in *chup1* or *chup1kac1kac2*. Additionally, we detected subtle but significant light-induced changes in the chloroplast distribution pattern on the uppermost mesophyll surface in *kac1kac2*, but not in *chup1* or *chup1kac1kac2*. Although our attempts to detect the light-induced chloroplast movement in *kac1kac2* under the microscope failed, we could detect the light-induced movement in *kac1kac2* through the measurement of leaf transmittance in which the sum of the subtle changes in the multiple cell layers is detectable.

In addition to *Arabidopsis*, a functional difference between CHUP1 and KAC was found in the moss *P*. *patens* [23–25]. The blue-light-induced chloroplast movement is dependent on both actin filaments and microtubules, and thus, is inhibited only when they are both disrupted by inhibitors [39]. Among the three *P*. *patens CHUP1* genes (*PpCHUP1A*, *B*, and *C*), *PpCHUP1A* is essential for actin-dependent chloroplast movement [23]. Because the structure and dynamics of short actin filaments on chloroplasts similar to *Arabidopsis* cp-actin filaments were found in *P*. *patens* [19], at least *Pp*CHUP1A is likely necessary for the regulation of these filaments in *P*. *patens*. The *PpCHUP1A* single and *PpCHUP1A/B* double knockout plants had impaired actin-dependent chloroplast movement but retained normal microtubule-dependent chloroplast movement, and they showed the normal attachment of chloroplasts to the plasma membrane [23]. Plants containing knockouts of two *P*. *patens KAC* genes (*PpKAC1* and *PpKAC2*) exhibited severe defects, such as no photorelocation movement and the strong aggregation of chloroplasts [24]. Although the complete aggregation of chloroplasts in the *PpKAC1/2* double knockout precluded the analysis of the involvement of *Pp*KACs in actin-filament- or microtubule-dependent chloroplast movement, the analyses of *PpKAC1/2* double RNAi lines revealed that *Pp*KACs primarily mediate actin-filament-chloroplast movement [25]. Therefore, CHUP1 and KACs coordinately regulate actin-filament-dependent chloroplast movement but also show some independent functions in land plants.

In *Arabidopsis*, CHUP1 and KACs exhibited quite distinct localization patterns although some parts of these proteins likely co-localized. When the functional fusion with florescent proteins were examined, CHUP1 localized on the chloroplast outer envelope [20, 21, 27, 34], whereas KAC localized primarily in the cytosol and partly on the plasma membrane [22, 40, 41]. A western blot analysis using fractionated protein showed the difference between CHUP1 and KACs; CHUP1 was detected in the microsomal fraction but KACs were detected primarily in the soluble fraction [16, 22, 40]. Nevertheless, KACs might mediate cp-actin-filament-dependent chloroplast movement only at the CHUP1-localized site, i.e., at the interface between the plasma membrane and the chloroplast where cp-actin filaments are found [9, 10], because CHUP1 is essential for KAC-dependent chloroplast movement.

### KAC-independent chloroplast photorelocation movement is mediated by phot2, CHUP1, PMI1, and THRUM1 and requires intact actin filaments

**Fig 6:**
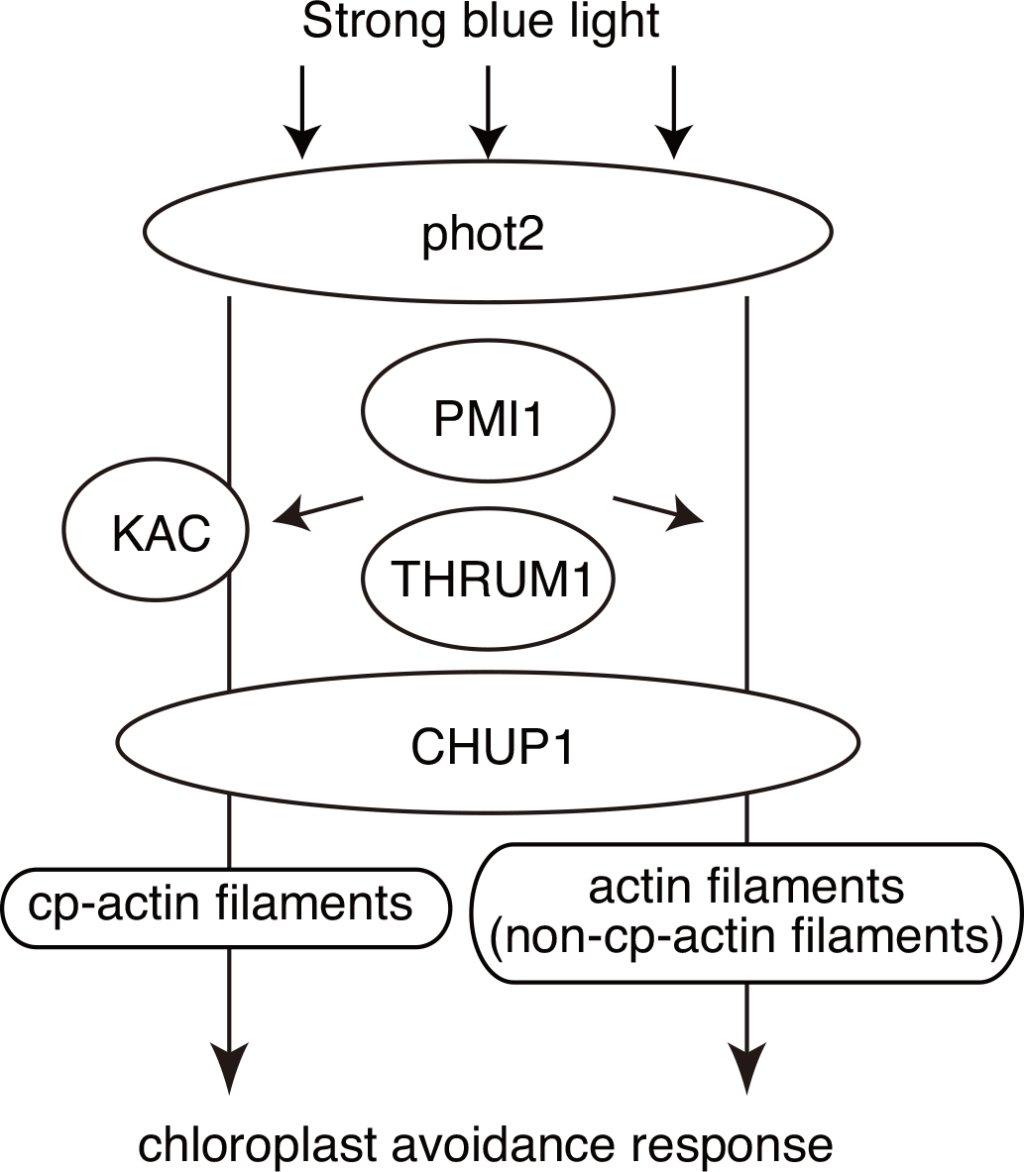
A model for strong-blue-light-induced avoidance response. In wild type, phot2 (and also phot1) perceive strong blue light and mediates cp-actin-filament-mediated avoidance response in CHUP1- and KAC-dependent manners. At least in *kac1kac2* mutant plants, light-induced chloroplast movement is induced in actin-filament- and CHUP1-dependent manners (but cp-actin-filament-independent). PMI1 and THRUM1 regulate both cp-actin-dependent or–independent responses, but the relationship between these proteins, CHUP1, and KAC is unclear.

The strong-light-induced chloroplast movement found in *kac1kac2* was severely attenuated in *phot2kac1kac2*, *chup1kac1kac2*, *pmi1kac1kac2*, and *thrum1kac1kac2*, indicating that phot2, CHUP1, PMI1, and THRUM1 are required for the KAC-independent chloroplast movement (Fig 6). Although phot2 is the main photoreceptor under the strong light condition, a very slight light-induced chloroplast movement was detected in *phot2kac1kac2* but not in *phot1phot2kac1kac2*, indicating that phot1 can mediate chloroplast movement in the absence of KACs, although inefficiently. Similarly, the phot1-dependent chloroplast avoidance response was observed in the *phot2* mutant in response to very strong white light [42]. Compared with phot1, phot2 localized on the chloroplast outer envelope at a higher amount [43]. Thus, irrespective of KAC-dependence, strong-blue-light-induced chloroplast movements might depend on phototropins localized on the chloroplast outer envelope.

Although CHUP1, PMI1, and THRUM1 are required similarly in the KAC-independent chloroplast movement, the mutant phenotypes of *chup1*, *pmi1*, and *thrum1* are quite different. The *chup1* mutant lacked any light-induced chloroplast movement (Fig 1) [10, 20]. Cp-actin filaments have not been detected in the *chup1* mutant under any light conditions [9–11]. Besides regulating cp-actin filaments, CHUP1 has the ability to associate with the plasma membrane through the coiled-coil region [21]. The CHUP1 N-terminal region, including the N-terminal chloroplast targeting signal sequence and the coiled-coil domain, is sufficient to mediate the attachment of chloroplasts to the plasma membrane, although the F-actin binding domain and the C-terminal region are implicated in the cp-actin filament regulation [21]. Thus, the connection between the chloroplast and the plasma membrane via the CHUP1 N-terminal region might play an important role in the KAC-independent chloroplast movement. Cp-actin filaments were not detected in *thrum1*, although *thrum1* exhibited a weak but significant chloroplast movement (both the accumulation and avoidance responses) (Fig 1)[10, 17]. THRUM1 has the ability to bundle actin filaments *in vitro* [17] and to interact with actin filaments *in vivo* [10,17]. Because THRUM1 interacts with both cp-actin filaments and cytoplasmic actin filaments, THRUM1 might mediate KAC-independent chloroplast movement through the interaction with cytoplasmic actin filaments. Consistent with a weak chloroplast photorelocation movement, cp-actin filaments were unstable and detected only under certain light conditions in *pmi1* mutants [16]. However, the biochemical function of PMI1 remained to be determined, and thus, it is difficult to imagine the role of PMI1 in KAC-independent and cp-actin-filament-dependent chloroplast movements.

### CHUP1 and KACs are indispensable for the nuclear avoidance response

The nuclear avoidance response is dependent on cp-actin-filament-mediated photorelocation movement of plastids in pavement cells and in mesophyll cells [16, 34]. The *chup1* mutant lacking cp-actin filaments was severely defective in the nuclear avoidance response but we detected a very subtle nuclear avoidance response in the *chup1* mutant. The *kac1kac2* mutant exhibited a weak but substantial nuclear avoidance response, and the *chup1kac1kac2* triple mutant exhibited no light-induced changes in the nuclear distribution pattern, indicating that CHUP1 and KACs redundantly mediate the nuclear avoidance response in pavement cells. When examined using microbeam irradiation, two types of light-induced nuclear movements were observed, phot2-dependent “avoidance movement” from the irradiated side towards the non-irradiated side, which depends on the cp-actin-filament-dependent movement of plastids attached to the nuclei, and the photosynthesis-dependent “parallel movement” along the actin bundles that are independent of the plastids [34]. The avoidance movement was attenuated in *kac1kac2*, and was abrogated in *chup1* and *chup1kac1kac2*, but the parallel movement was enhanced in these mutant plants compared with in wild type, indicating that the parallel movement is independent of CHUP1 and KAC. Compared with *chup1* single mutant plants [34], the proportion of the parallel movement was slightly decreased in *chup1kac1kac2* and, consequently, the proportion of nuclei unresponsive to light was higher in *chup1kac1kac2*. Because KAC1 shows actin-binding activity [22], the KACs might mediate parallel movement through the actin-binding, at least in the absence of CHUP1. Interestingly, approximately half of the nuclei are in the light position irrespective of the light condition. This phenotype is highly similar to that of the *pmi1pmir1* double and *pmi1pmir1pmir2* triple mutant plants [16], indicating that CHUP1, KAC, and PMI1/PMIR1 should function at a similar step. The *phot2* mutant also exhibited no light-induced changes in the nuclear distribution pattern, but only approximately 30% of the nuclei were in the light position regardless of the light condition, which was similar to dark-adapted wild type plants (70% in the dark position) [34], indicating that CHUP1, KAC, and PMI1/PMIR1 mediate nuclear positioning independent of phot2 in the darkness. Although cp-actin filaments were not detected on pavement plastids in *chup1* and *pmi1pmir1pmir2* mutant plants [16, 34], *phot2* retained cp-actin filaments on the pavement plastids [34]. Therefore, cp-actin filaments should be required for the nuclear positioning on the cell bottom in darkness.

## Conclusion

We revealed that CHUP1 and KAC proteins cooperatively, but somewhat independently, function during strong-blue-light-induced movements of chloroplasts and nuclei. Although cp-actin filaments are required for the fast directional movements of chloroplasts [9–11], our present findings indicate that another actin-dependent mechanism control light-induced changes in chloroplast positioning independently of cp-actin filaments. This cp-actin filament-independent mechanism still be dependent on the factors involved in the regulation of cp-actin filaments, i.e., CHUP1, PMI1, and THRUM1. Together with phototropin and PMI1/PMIR, CHUP1 and KAC are highly conserved factors in Streptophytes and thus these proteins are core factors for chloroplast movement in Streptophytes [44]. Unraveling the molecular properties of these factors is required for understanding the molecular mechanism.

## Supporting Information

S1 Fig Chloroplast photorelocation movement induced by strong blue light in mutant plants. **(a, b)** Changes in leaf transmittance rates from 2 to 6 min after changes in light fluence rate (3 and 20 μmol m^–2^ s^–1^) are indicated as percentage transmittance change over 1 min. Data for **(a)** in *chup1*, *pmi1*, and *thrum1* backgrounds and **(b)** in the phototropin mutant background were derived from Fig 1B, 1C, 1D and 1E, respectively. **(c-e)** KAC-independent chloroplast movement was analyzed in *jac1*, *web1*, and *pmi2pmi15* backgrounds. Mean values from three independent experiments are shown. Error bars indicate standard errors.
(PDF)
