# Correction: Light-Induced Movements of Chloroplasts and Nuclei Are Regulated in Both Cp-Actin-Filament-Dependent and -Independent Manners in *Arabidopsis thaliana*

**DOI:** 10.1371/journal.pone.0168318

**Published:** 2016-12-08

**Authors:** Noriyuki Suetsugu, Takeshi Higa, Eiji Gotoh, Masamitsu Wada

There are errors in the third and fourth sentences of the second paragraph under the subheading “KAC proteins and CHUP1 redundantly mediate the nuclear avoidance response in pavement cells” in the Results section. The correct sentences are: The *chup1* mutant had severely attenuated the avoidance movements and the parallel movement was prominent in *chup1*, indicating that cp-actins existed on the plastids attached to the nuclei, which is essential for the avoidance response and to suppress parallel movements (Fig 5A; data from [34]; chi-square test, *P* < 0.0005 for wild type vs. *chup1*). The *kac1kac2* was similar to wild type (chi-square test, P > 0.1 for wild type vs. *kac1kac2*), but only approximately 20% of the nuclei showed the avoidance movement (Fig 5A and 5B).
