# Supplementary figures and images for: Light-Induced Movements of Chloroplasts and Nuclei Are Regulated in Both Cp-Actin-Filament-Dependent and -Independent Manners in Arabidopsis thaliana
Source: PLoS One. 2016 Jun 16;11(6):e0157429. doi: 10.1371/journal.pone.0157429 (PMC4911103; doi:10.1371/journal.pone.0157429)

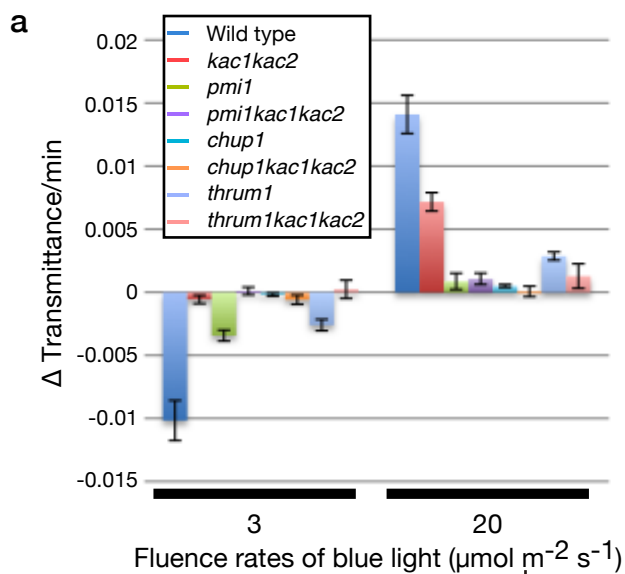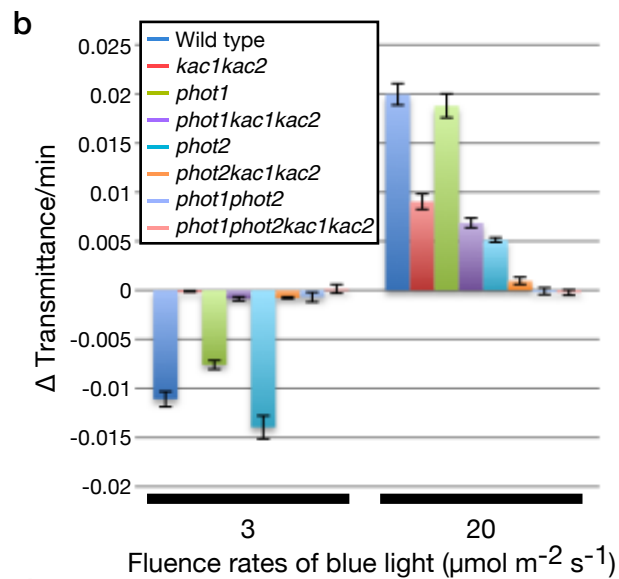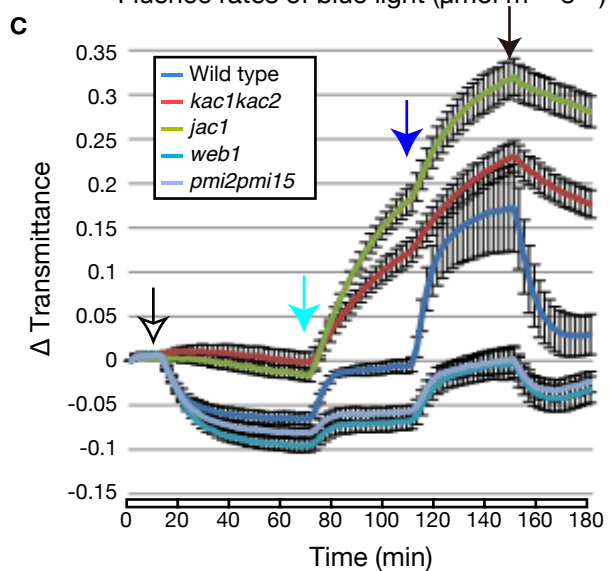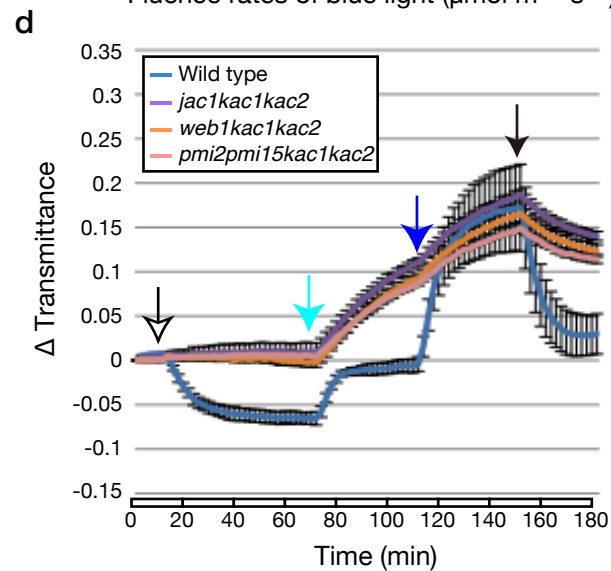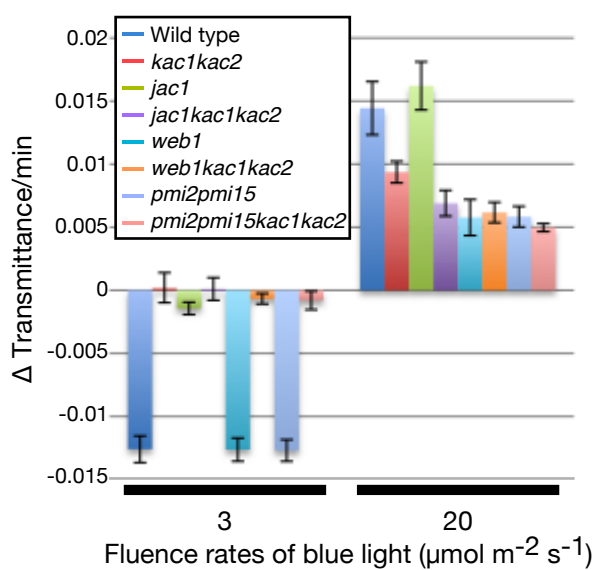

Supplement: S1 Fig — (a, b) Changes in leaf transmittance rates from 2 to 6 min after changes in light fluence rate (3 and 20 μmol m–2 s–1) are indicated as percentage transmittance change over 1 min. Data for (a) in chup1, pmi1, and thrum1 backgrounds and (b) in the phototropin mutant background were derived from Fig 1B, 1C, 1D and 1E, respectively. (c-e) KAC-independent chloroplast movement was analyzed in jac1, web1, and pmi2pmi15 backgrounds. Mean values from three independent experiments are shown. Error bars indicate standard errors. (PDF) [file pone.0157429.s001.pdf]
